# ‘A series of unfortunate events’: a case report of infective endocarditis resulting from ventricular arrhythmia ablation

**DOI:** 10.1093/ehjcr/ytad604

**Published:** 2023-11-27

**Authors:** Maria Stec, Dominika Dziadosz, Katarzyna Mizia-Stec

**Affiliations:** First Department of Cardiology, School of Medicine in Katowice, Medical University od Silesia, Ziołowa 47, 40-635 Katowice, Poland; First Department of Cardiology, School of Medicine in Katowice, Medical University od Silesia, Ziołowa 47, 40-635 Katowice, Poland; First Department of Cardiology, Upper Silesian Medical Centre, School of Medicine in Katowice, Medical University of Silesia, 40-055 Katowice, Poland; First Department of Cardiology, Upper Silesian Medical Centre, School of Medicine in Katowice, Medical University of Silesia, 40-055 Katowice, Poland

**Keywords:** Infective endocarditis, Ventricular tachycardia, Pseudoaneurysm, Radiofrequency ablation, Case report

## Abstract

**Background:**

Radiofrequency ablation (RFA) is the most effective non-pharmacological approach in the reduction of ventricular tachycardia (VT) recurrence. However, it is crucial to thoroughly screen every patient for contraindications for RFA and provide appropriate pharmacological prophylaxis, if needed, since adverse effects may be fatal.

**Case summary:**

A 77-year-old male with multi-vessel coronary artery disease, heart failure with reduced ejection fraction (New York Heart Association (NYHA) Class III), with implantable cardioverter-defibrillator was admitted to our clinic due to recurrent life-threatening VT. The patient presented several concomitant diseases: dyslipidaemia, hypertension, and chronic kidney disease in Stage IIIB. He had a history of two myocardial infarctions and coronary artery bypass grafts complicated by mediastinitis and dehiscence of a sternotomy scar (2013). Radiofrequency ablation and pace mapping of VT were performed in sterile conditions, but no pre-operative antibiotic prophylaxis was administered. There were no local or general complications in the post-operative period. The patient was discharged from the clinic in good condition. A week later, the patient suffered from septic shock and infective endocarditis of mitral valve complicated with infiltration of the ventricular septum, wall dissection of the left ventricle (LV), pseudoaneurysm, and abscess of the LV. At the time of the second hospitalization extensive dental carries were found and oral cavity sanitation was performed. Due to the severity of the condition, patient did not survive.

**Conclusion:**

Oral cavity infections are common but often overlooked, mainly when the RFA procedure is urgent. A thorough physical examination, including a dental check-up, is crucial to minimize the risk of potential infection of the endocardial tissue and maximize the benefits of the therapy. Still, it is possible that the myocardial infection was not a result of oral cavity infection but a result of other undiagnosed and untreated infection. Contamination of the procedure site with patients’ own microbiota or foreign microorganisms by the medical personnel is also a likely and unfortunate scenario. The presented case highlights the significance of not only prophylaxis, screening, and treatment of possible inflammation sites before RFA but also the need for sustaining sanitary standards and sterile conditions.

**Learning points**

- Prevention of infection and antibiotic prophylaxis is crucial to ensure the safety of high-risk and low-risk patients undergoing transcatheter procedures.
- Thorough screening and elimination of all possible inflammation sites before any radiofrequency ablation are essential to minimize the risk of potential infection of the endocardial tissue.
- Dental carries were the most probable origin of the infection.
- In this case, radiofrequency ablation of left ventricular arrhythmia was considered a lifesaving procedure. Unusual presentation of infective endocarditis was a result of the infection of the ablated region of endocardium. Necrotic tissue and local thrombosis secondary to the ablation could have been additional triggers for this process.
- Dissection and abscess of the left ventricle wall constitute a rare and severe presentation of infective endocarditis.

## Introduction

Radiofrequency ablation (RFA) is considered to be the most effective non-pharmacological approach in the reduction of recurrence of an electrical storm (ES) due to sustained monomorphic ventricular tachycardia (VT) refractory to anti-arrhythmic drugs with a recommendation of Class IB.^1^ We hereby report a case of infective endocarditis (IE) of mitral valve complicated with left ventricular (LV) dissection, pseudoaneurysm, and abscess.

## Summary figure

**Figure:**
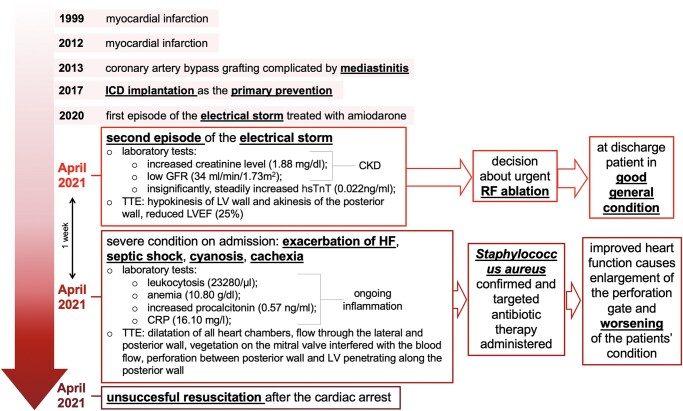


## Case presentation

We present a case of a 77-year-old male burdened by a multi-vessel coronary artery disease, heart failure with reduced ejection fraction (EF) of 20–25%, with implanted dual-chamber implantable cardioverter-defibrillator (ICD) for primary prevention admitted to our clinic due to recurrent life-threatening VT. The patient presented the following concomitant diseases: dyslipidaemia, hypertension, and chronic kidney disease (CKD) in Stage IIIB. He had a history of two myocardial infarctions and coronary artery bypass graft (CABG) of the left internal mammary artery and the left anterior descending artery (LIMA-LAD), right internal mammary artery and the right coronary artery (RIMA-RCA), and aorta-obtuse marginal artery (Ao-OM). Coronary artery bypass graft was complicated by mediastinitis and dehiscence of a sternotomy scar (2013).

Moreover, in 2013, the patient suffered from sigmoid colon necrosis, which resulted in sigmoidectomy with a colostomy. In 2017, a patient had a biventricular ICD implanted. In 2020, a patient had the first episode of an ES. The patient has been vaccinated against severe acute respiratory syndrome coronavirus 2 with two doses of the vaccine.

In May of 2021, the patient presented to our clinic’s emergency room. With recurrent symptomatic, haemodynamically stable VT (150 b.p.m.) after two shock deliveries. On physical examination, the patient was alert and oriented. A sternotomy scar with telangiectasias and colostomy was observed. There were no other pathological findings regarding the respiratory, gastrointestinal, musculoskeletal, or nervous system.

On admission, electrocardiogram (ECG) revealed ICD DDD stimulation with a pace of 70 b.p.m. Data obtained from the ICD device had shown several episodes of VT and anti-tachycardia pacing (ATP) therapy and two shock deliveries. Laboratory tests did not reveal any abnormalities, except for the raised level of creatinine (1.88 mg/dL, *n* = 0.1–1.3) with a glomerular filtration rate (GFR) of 34 mL/min/1.73 m^2^ (*n* = 90–120) and a slightly raised level of high-sensitive cardiac troponin T (0.022 ng/mL, *n* = 0–0.014). Severe acute respiratory syndrome coronavirus 2 rapid antigen test was negative.

**Figure 1:**
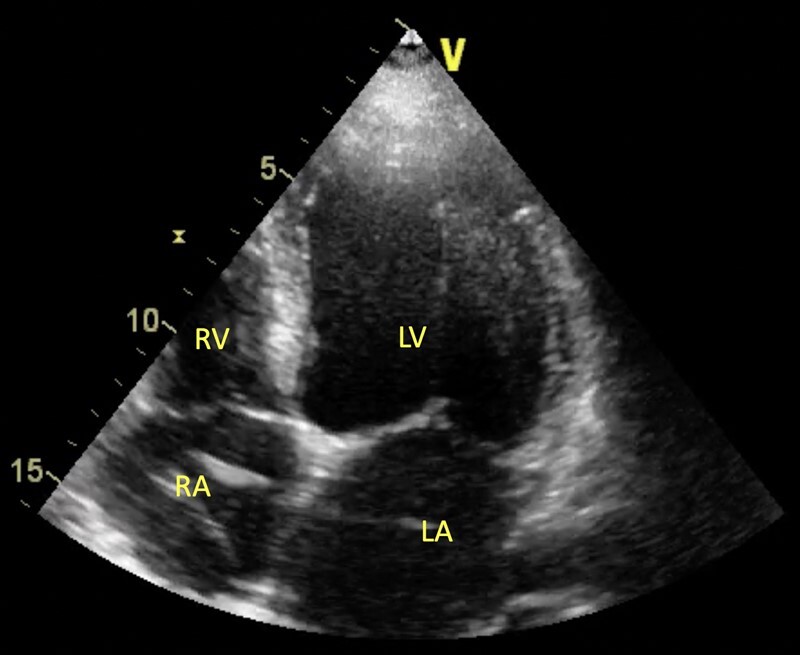
Preliminary scheme. Transthoracic echocardiography, four-chamber view. On admission during the first hospitalization in 2021. No pathological abnormalities visible. LA, left atrium; LV, left ventricle; RA, right atrium; RV, right ventricle.

Transthoracic echocardiography (TTE) on admission has shown global hypokinesis of the LV, akinesis of the posterior and inferior wall with LVEF of 20–25% (*Figure 1*). Pericardial effusion was not observed.

During hospitalization, the patient presented with an additional incidence of VT ceased by ATP therapy. Moreover, the patient underwent planned coronary angiography that has shown insignificant 40% RCA stenosis, 60% LAD stenosis, open LIMA-LAD bypass, and non-open RIMA-RCA and Ao-OM bypass. After the procedure, exacerbation of the CKD was observed, with a creatinine level of 2.04 mg/dL and an estimated GFR (eGFR) of 31 mL/min/1.73 m^2^. Due to the worsening of kidney function, the pharmacotherapy was modified, and eplerenone and telmisartan were discontinued. After the improvement of eGFR, we decided to escalate the HF treatment. Sacubitril-valsartan was implemented (24/26 mg *b.i.d.*).

Following the diagnostic path, the patient was qualified for ablation and pace mapping of the LV with a 3D electroanatomic system (Carto3). The procedure was performed with a trans-septal and retrograde approach. It has shown a vast low-voltage area of the inferior wall, posterior wall, and ventricular septum. With the use of pace mapping, RFA was applied from the apical to the basal region in the low-voltage area. The procedure was executed without any complications. In the post-operative period, unfractionated heparin was supplemented for 24 h according to the current European Society of Cardiology (ESC) guidelines. However, one day after, the patient had a recurrence of VT treated with amiodarone.

The patient had been discharged from the clinic, in good general condition, without ongoing arrhythmias and DDD stimulation of 80 b.p.m., with a plan of ICD to cardiac resynchronization therapy defibrillator upgrade.

A week after the discharge from the clinic, the patient returned to our clinic’s emergency room. The patient was in a severe condition, with signs of acute decompensated heart failure, cyanosis, and oliguria. Laboratory tests revealed leucocytosis of 24.46 10^3^/µL (*n* = 4.5–11), anaemia–haemoglobin concentration of 9.80 g/dL (*n* = 13.8–17.2), and erythrocyte count of 3.22 10^6^/µL (*n* = 4.7–6.1). Elevated CRP of 224 mg/L (*n* ≤ 1.0) and procalcitonin of 0.57 ng/mL (*n* ≤ 0.05) indicated an ongoing inflammation process, with a creatinine level of 2.19 mg/dL and GFR of 29 mL/min/1.73 m^2^. Transthoracic echocardiography revealed dilatation of all cardiac chambers with LVEF of 20% and segmental contractility disturbances (akinesis of inferior and posterior LV wall and hypokinesis of intraventricular septum).

**Figure 2:**
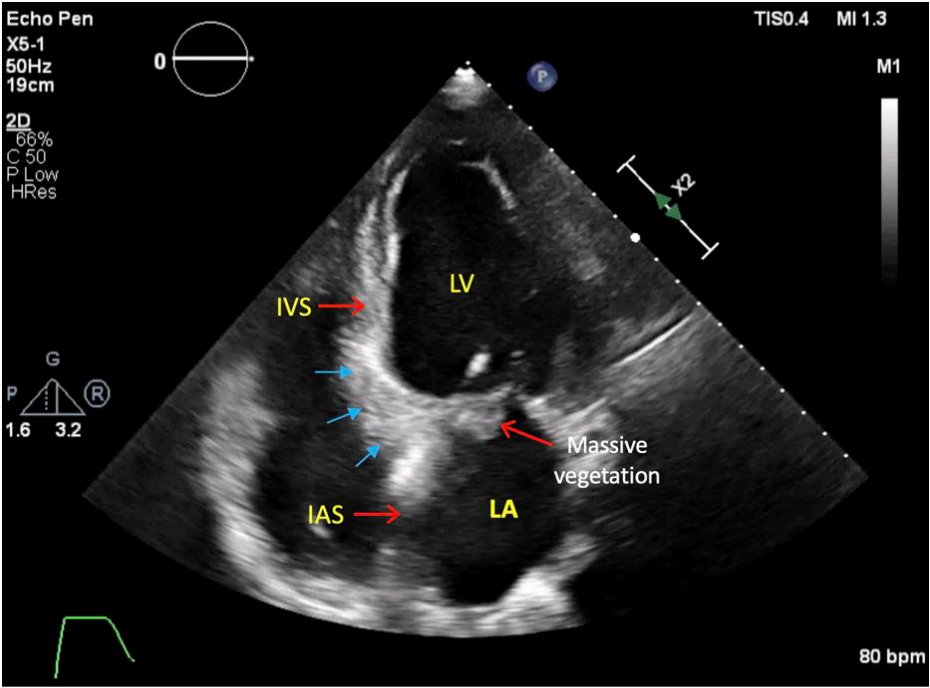
Transthoracic echocardiography, four-chamber view. On admission during the second hospitalization in 2021. Blue arrows point on thickening of the interatrial and interventricular septum. IAV, interatrial septum; IVS, interventricular septum; LA, left atrium; LV, left ventricle.

**Figure 3:**
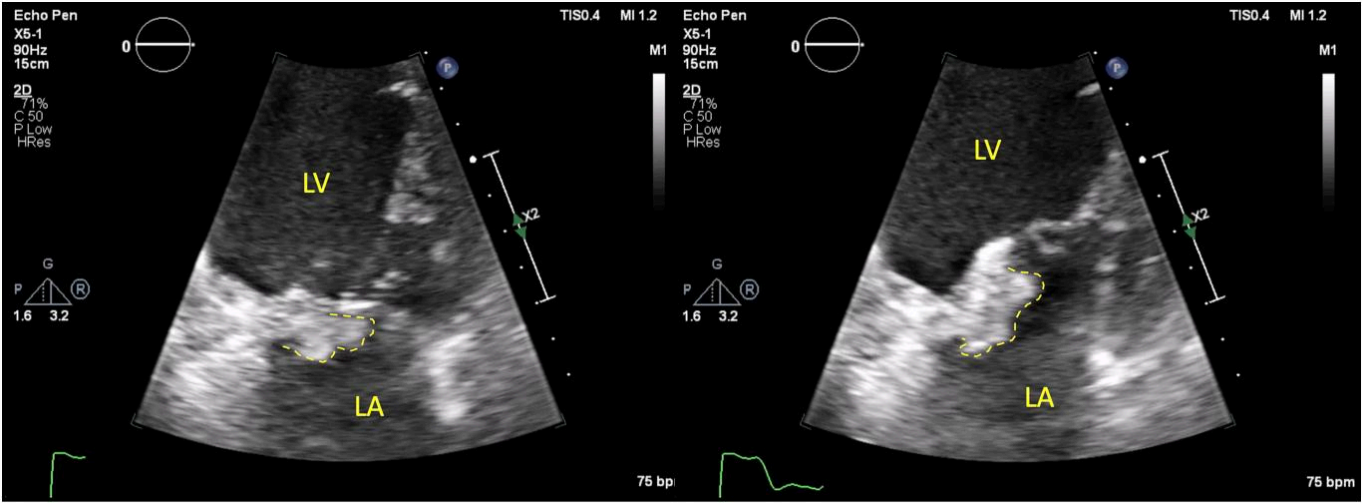
Transthoracic echocardiography, two-chamber view, zoom. On admission during the second hospitalization in 2021. Yellow line shows vegetation on leaflets of the mitral valve. LA, left atrium; LV, left ventricle.

Moreover, 13 × 21 mm vegetation was visible on the A2 segment of the mitral anterior leaflet without any mitral regurgitation (*Figures 2* and *3*). Transthoracic echocardiography revealed also unusual features of IE: increased thickness of inferior LV wall and thickening of interatrial and interventricular septum measured up to 12 mm (*Figure 2*). Bacteraemia was confirmed with blood samples positive for *Staphylococcus aureus*. The patient was qualified for elective treatment with broad-spectrum antibiotics (vancomycin 1.00 g i.v. *b.i.d.* and gentamycin 180 mg i.v. *o.d.*).

In the following days, inflammatory markers presented an increasing tendency. Thus, we were seeking possible inflammation sites. Dental caries have been found. Oral cavity sanitation was performed.

**Figure 4:**
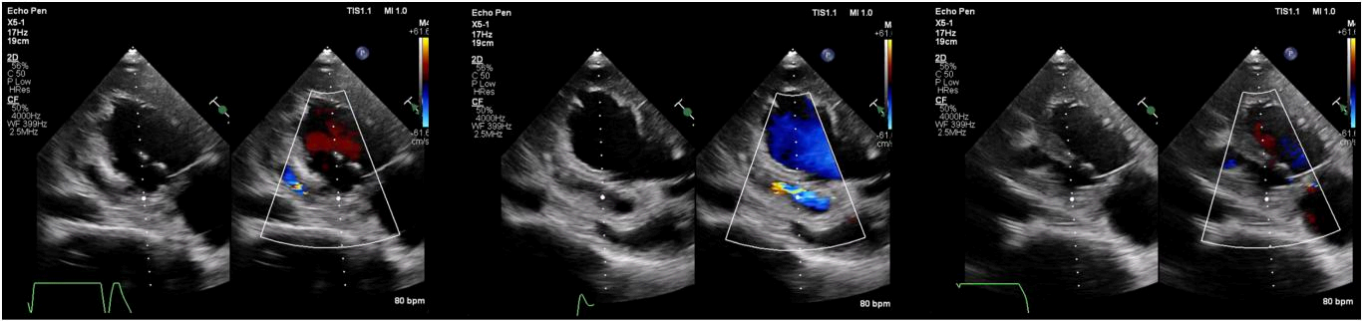
Transthoracic echocardiography, colour Doppler imaging/colour compare view. During the second hospitalization in 2021. Left image: apical two-chamber view, dissection and perfusion of left ventricular wall; middle image: apical three-chamber modified view, dissection and perfusion through left ventricular wall; right image: apical two-chamber view, ostium of dissection and pseudoaneurysm.

**Figure 5:**
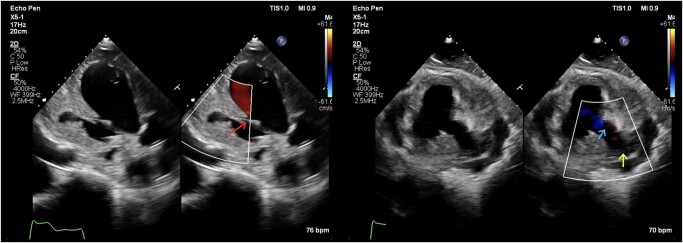
Transthoracic echocardiography, colour Doppler imaging/colour compare view. During the second hospitalization in 2021. Left image: apical three-chamber view, portal of dissection (red arrow) and pseudoaneurysm; right image: parasternal short axis, mid-cavity view, portal of dissection, long neck of pseudoaneurysm (blue arrow) and pseudoaneurysm cavity (yellow arrow).

Subsequent TTE monitoring during hospitalization revealed further extension of IE pathology. It has shown wall dissection with pseudoaneurysm measured as 54 × 40 mm and bidirectional blood flow (*Figures 4* and *5*).

Pericardial effusion and hydrothorax with a maximal fluid level of 7 cm in the left basal region were observed.

Cardiac computed tomography (CT) showed hydrothorax of both lungs with atelectasis, pseudoaneurysm of the LV, and pericardial effusion of 14 mm around it. Further contrast imaging (CT or cardiac magnetic resonance (CMR)) was not performed because of the progression of CKD. The patient was disqualified from the cardiothoracic procedure due to the lack of cardiac surgery option and elevated inflammation markers. Two days later, the patient suffered from a cardiac arrest. Pulseless electrical activity was confirmed. Cardiopulmonary resuscitation (CPR) and intubation were performed. Sadly, the patient passed away.

## Discussion

Electrical storm has been defined as ≥3 episodes of sustained ventricular arrhythmia (VA) occurring within 24 h, requiring either ATP or cardioversion/defibrillation, with each event separated by at least 5 min. The severity of ES may vary from asymptomatic VT episodes terminated by ATP to a life-threatening electrical instability with VA that reappears frequently, even after multiple shocks.^1,2^ Choice of therapy depends on the type of VA and underlying aetiology. Radiofrequency ablation is the most effective non-pharmacological approach in the reduction of recurrence of ES due to incessant slow or sustained monomorphic VT refractory to anti-arrhythmic drugs. It should be considered in patients with recurrent symptomatic episodes of polymorphic VT or VF triggered by a similar premature ventricular complex.^1^ Successful ablation is thought to reduce relapse of ES or VT episodes and improve survival in retrospective studies.^3,4^

In the presented case, IE could have been triggered by RFA due to an ongoing inflammation of the oral cavity at the time of ablation. It is key to mention that the patient did not receive antibiotic prophylaxis. The presented case highlights the importance of screening for the inflammation sites before RFA in all patients to minimize the risk of potential infection of the endocardial tissue and maximize the benefits of the therapy.

However, the current ESC guidelines regarding IE^5^ suggest only limited preventive measures in prophylaxis of IE. Guidelines mention only three categories of patients with the highest risk of IE:

- patients with a prosthetic valve or with prosthetic material used for cardiac valve repair, transcatheter-implanted prostheses, and homografts;
- patients with previous IE; and
- patients with untreated cyanotic congenital heart disease (CHD) and those with CHD who have post-operative palliative shunts, conduits, or other prostheses.

Our patient did not fit the above-mentioned criteria and was neither classified nor treated as the highest-risk patient. The current guidelines specifically emphasize the elimination of possible dental infection sources at least 2 weeks before the elective procedure. Additionally, screening of nasal carriage for the presence of *S. aureus* before the surgery is also recommended prior to a scheduled and not urgent procedure.^6,7^ Furthermore, the ESC task force does not recommend antibiotic prophylaxis in patients with an intermediate risk of IE. Nevertheless, it is key to bear in mind that all sites of inflammation stimulate arrhythmias; therefore, the elimination of inflammation sites may reduce the risk of arrhythmia or IE. In the presented case, RFA was a lifesaving procedure with no additional time to eliminate possible sources of dental sepsis. The negative outcome of the patient magnifies the need for establishing clear guidelines for prophylaxis before acute procedures to increase the chance of patient’s.

The presented case points out that we cannot overlook patients from intermediate or low risk of IE or without enlisted risk factors and that a thorough search for inflammation is equally important in all patients.

It may be seen in the described patient, with cachexia hence with immunodeficiency, that even minuscule site of inflammation such as dental caries might have caused inflammation of the ablated region alongside mural thrombus, which later escalated to sepsis.

Origin of microbiota in IE varies from bacteria of the skin, gastrointestinal tract, to oral cavity microbiota.^8^ According to Murdoch *et al*., the most common cause of IE is *S. aureus*, which can be found both in microbiota of the skin and oral cavity.^9,10^ A common and often omitted source of inflammation and bacteraemia is an oral cavity, especially dental caries.^11^ Therefore, regular dental screening and treatment should be emphasized by cardiologists in their day-to-day practice.

Although RFA of VAs is a safe and prevalent procedure, even its least common complications need to be noted, especially in patients with a history of inflammatory complications. Distinct attention should be given to the prevention of intraprocedural equipment contamination and post-procedural hygiene, considering it may result in the colonization of *S. aureus*.^12^ Establishing and maintaining sanitary standards in medical centres, especially operating facilities, are crucial to provide safe and hygienic conditions for every elective, urgent, and lifesaving procedure.

Currently, there are little data regarding routine antibiotic prophylaxis for every patient undergoing RFA. Even though it could help minimize the risk of infection, it has its downsides, like microbial resistance, which poses a challenge for healthcare worldwide.

This clinical case shows the need for further research regarding antibiotic prophylaxis for safe cardiac transcatheter procedures and is a reminder to uphold what is already known, which is the importance of sanitation and hygiene in healthcare facilities.

## Supplementary Material

ytad604_Supplementary_Data

## Data Availability

The data underlying this article will be shared on reasonable request to the corresponding author.

## Lead author biography

I am a sixth-year medical student at the Medical University of Silesia in Katowice, Poland. As a young scientist, my interests oscillate around cardiology with a special focus on echocardiography and imaging. Despite being at the beginning of the career path, I had the pleasure of taking part in numerous scientific fellowships and research grants including an internship at the Zayed Centre for Research into Rare Diseases in Children, UCL, London and Karolinska Institutet.

## Notes

**Consent:** The authors confirm that written consent, in line with COPE guidelines, for submission and publication of this case report including images and associated text has been obtained from the patient.

**Funding:** None declared.
